# The effect and mechanism of traditional Chinese exercise for chronic low back pain in middle-aged and elderly patients: A systematic review

**DOI:** 10.3389/fnagi.2022.935925

**Published:** 2022-10-10

**Authors:** Xue-Qiang Wang, Huan-Yu Xiong, Shu-Hao Du, Qi-Hao Yang, Li Hu

**Affiliations:** ^1^Key Laboratory of Mental Health, Institute of Psychology, Chinese Academy of Sciences, Beijing, China; ^2^Department of Psychology, University of Chinese Academy of Sciences, Beijing, China; ^3^Department of Sport Rehabilitation, Shanghai University of Sport, Shanghai, China

**Keywords:** chronic low back pain, traditional Chinese exercise, elderly people, Tai Chi, Qigong

## Abstract

**Background:**

Increasing lines of evidence indicate that traditional Chinese exercise (TCE) has potential benefits in improving chronic low back pain (CLBP) symptoms. To assess the clinical efficacy of TCE in the treatment of CLBP, we performed a systematic review of existing randomized controlled trials (RCTs) of CLBP and summarized the neural mechanisms underlying TCE in the treatment of CLBP.

**Methods:**

A systematic search was conducted in four electronic databases: PubMed, Embase, the Cochrane Library, and EBSCO from January 1991 to March 2022. The quality of all included RCTs was evaluated by the Physiotherapy Evidence Database Scale (PEDro). The primary outcomes included pain severity and pain-related disability.

**Results:**

A total of 11 RCTs with 1,256 middle-aged and elderly patients with CLBP were included. The quality of all 11 included RCTs ranged from moderate to high according to PEDro. Results suggested that TCE could considerably reduce pain intensity in patients with CLBP. Overall, most studies did not find any difference in secondary outcomes (quality of life, depression, and sleep quality).

**Conclusion:**

The neurophysiological mechanism of TCE for treating CLBP could be linked to meditation and breathing, posture control, strength and flexibility training, and regulation of pain-related brain networks. Our systematic review showed that TCE appears to be effective in alleviating pain in patients with CLBP.

## Introduction

Incidence of low back pain (LBP) increases progressively with age (Neuhauser et al., 2005); it is estimated that 12% of adults over the age of 65 suffer from chronic LBP (CLBP) (Arnstein, 2010). When LBP in older population becomes chronic (lasting more than 12 weeks) (Deyo et al., 2014), it can lead to a variety of harmful consequences, including falls and fractures (Leveille et al., 2009), depression/anxiety (Meyer et al., 2007; Kroenke et al., 2013), social difficulties (Mackichan et al., 2013), and sleep disturbances (Weiner et al., 2006a). In addition, extraspinal conditions (i.e., osteoarthritis and fibromyalgia) are common in older adults with CLBP and may be linked to pain-related disability (Weiner et al., 2006b; Viniol et al., 2013; Rundell et al., 2017). Although clinicians have treated CLBP with conventional medication and surgery for a long time, many patients continue to experience pain without significant pain relief (Steffens et al., 2016; Maher et al., 2017). Therefore, over the past 30 years, many clinical guidelines have recommended that treatment of CLBP should focus on non-pharmacological treatments, such as exercise therapy and mind–body exercise (Bernstein et al., 2017; Qaseem et al., 2017; Wong et al., 2017; Stochkendahl et al., 2018; Zhang et al., 2019; Peng et al., 2022; Xiong et al., 2022). Exercise therapy, which includes a variety of interventions ranging from aerobic exercise to muscle strength training, has been shown to be useful in alleviating pain (Lawand et al., 2015; Wieland et al., 2017; Russo et al., 2018; Smith et al., 2022; Wu et al., 2022).

Under this condition, traditional Chinese exercise (TCE), as a therapeutic mind–body exercise, has been widely concerned by researchers (Koh, 1982; Chou et al., 2015; Guo et al., 2018). TCE [i.e., Tai Chi (Zou et al., 2017a) and Qigong (Zou et al., 2018a)] is becoming increasingly popular around the world and is being used to treat various diseases and prevent chronic disease progression (Zhu et al., 2016). TCE emphasizes mind–body integration; slow body movements should be synchronized with musculoskeletal relaxation, respiratory control, and mental focus in a meditative state (Luo et al., 2017; Zou et al., 2018c). In addition, TCE requires the stability of the trunk muscles to maintain the center of gravity, which embodies the principle of core stability training (Wang et al., 2013). In recent years, TCE has been successfully used worldwide for the treatment of CLBP and is recommended as a therapeutic activity according to the guidelines of the American College of Physicians (Qaseem et al., 2017). A meta-analysis also suggested that TCE might provide some pain relief in patients with LBP(Zhang et al., 2019). For instance, Blodt et al. (2015) suggested that Qigong training was no worse than exercise therapy for pain relief in patients with CLBP. Our previous work also supported that the patients with chronic non-specific LBP over the age of 50 engaging in Chen-style Tai Chi for 12 weeks had significantly reduced pain (Liu et al., 2019; Zou et al., 2019a). However, results from different randomized controlled trials (RCTs) are inconsistent, with some studies suggesting that yoga and Qigong had no effect on relieving CLBP possibly due to the small sample size or differences in pain sensitivity and processing in the elderly (Teut et al., 2016). The conclusions from current studies have remained controversial. In addition, there are no systematic reviews of TCE interventions for CLBP in the middle-aged and elderly. Therefore, further review and analysis of available data on TCE-related pain and disability in middle-aged and elderly patients with CLBP are necessary.

## Materials and methods

### Search strategy and inclusion criteria

This systematic review was registered with the Open Science Framework (10.17605/OSF.IO/NWGSF).^1^ PRISMA guidelines were followed (Moher et al., 2009). PubMed, Embase, the Cochrane Library, and EBSCO were searched from January 1991 to March 2022 for relevant clinical trials (Supplementary material 1). The following combination of terms was used as search keywords in the title and abstract: T1 = Tai Chi OR “Tai Chi *” OR Qigong OR Liuzijue OR Wuqinxi OR Yijinjing OR Baduanjin OR “traditional exercise” OR traditional Chinese medicine OR “Chinese traditional exercise” OR “traditional Chinese exercise” OR “Chinese exercise,” T2 = back pain OR low backache OR lower back pain OR lumbago OR lumbosacral pain OR sciatica. When screening clinical trials, the inclusion criteria are as follows:

(1) Types of studies. We included only published articles from RCTs that examined the effect of TCE on LBP. The article language was limited to English.
(2) Participants. All middle-aged and elderly patients (mean age > 35 years old) with a diagnosis of LBP were considered for this review.
(3) Interventions. The interventions included different types of TCE (i.e., Tai Chi, Baduanjin, Yijinjing, Qigong, Liuzijue, and Wuqinxi). Clinical trials comparing TCE with no intervention, placebo (waiting-list, unaltered lifestyle), or other treatments (such as exercise therapy, massage, and physical activity) were included.
(4) Types of outcome measures. Outcome measures should include at least one of two evaluations: pain and disability.

### Study selection and data extraction

Two authors independently screened all titles, abstracts, and main text of the relevant papers according to the inclusion criteria. Papers that did not match the criteria for inclusion were omitted. Disagreements were settled by discussion or a third reviewer. The following information was extracted from the selected articles: (1) published data (author, year); (2) design of included studies (subject subgroup, sample size, randomization, follow-up, clinical outcome measures, and time points); (3) type of intervention (including dose regimen, duration); (4) characteristics of participants (including baseline demographic information and diagnostic/inclusion/exclusion criteria); and (5) adverse effects.

### Quality assessment and data analysis

We used the Physiotherapy Evidence Database scale (PEDro) to assess the risk of bias for inclusion and the methodological quality of each study in this systematic review (Supplementary Table 1). Two authors independently evaluated the quality of the included RCTs, and all disagreements were settled by discussion or a third reviewer. The following information was evaluated: randomized allocation, concealed allocation, baseline comparability, blind subjects, blind therapists, blind assessors, adequate follow-up, intention-to-treat analysis, between-group comparisons, point estimates, and variability. Scores < 4 points were considered as poor quality; 4–5 points as modern quality; 6–8 points as high quality; 9–10 as excellent quality. The characteristics of the included RCTs were examined (Table 1). Then, The findings were then narratively presented in terms of the TCE’s mechanisms in the treatment of LBP, which were detailed and discussed in the following sections.

**TABLE 1:** Summary of included studies.

References	Country	Participant characteristic, sample size	Disease	Drugs	Intervention	Time point	Duration of trial period	Primary Outcomes	Result
Hall et al., 2011	Australia	160 subjects *M* = 41, *F* = 119 Mean age (± SD): 44.4 ± 13.2	Persistent low back pain	NA	G1 (*n* = 80): Tai Chi G2 (*n* = 80): Control group(usual health care)	10 weeks	18 sessions over 10 weeks (2 times per week for 8 weeks followed by once per week for 2 weeks)	1. Pain intensity (NRS) 2. Disability (RMDQ)	Tai Chi produced greater reductions in pain symptoms and pain-related disability than the control intervention.
Weifen et al., 2013	China	320 subjects *M* = 192, *F* = 128 Mean age (± SD): 37.6 ± 5.4	Chronic non-specific low back pain	NA	G1 (*n* = 141): Tai Chi group G2 (*n* = 47): Backward walking group G3 (*n* = 47): Jogging group G4 (*n* = 38): Swimming group G5 (*n* = 47): No exercise group	6 months	G1: Five 45 min sessions per week for 6 months G2-5: Five 30 min sessions per week for 6 months	1. Pain intensity (NRS)	After three and six months, no statistically significant difference in the intensity of LBP was demonstrated between the tai chi and swimming groups; significant differences were demonstrated among the tai chi and backward walking, jogging, and no exercise groups.
Blodt et al., 2015	Germany	127 subjects *M* = 25, *F* = 102 Mean age (± SD): 46.7 ± 10.4	Chronic non-specific low back pain	No medication taken during the period of study	G1 (*n* = 64): Qigong group G2 (*n* = 63): Exercise therapy group	3 months	Weekly sessions of 90 min over a period of 3 months	1. Pain intensity (VAS)	Qigong was not proven to be non-inferior to exercise therapy in the treatment of chronic LBP.
Teut et al., 2016	Germany	176 subjects *M* = 20, *F* = 156 Mean age (± SD): 73 ± 5.6	Chronic non-specific low back pain	No medication taken during the period of study	G1 (*n* = 61): Yoga group G2 (*n* = 58): Qigong group G3 (*n* = 57): Control group (no additional intervention)	3 months	1. Yoga (24 classes, 45 min each, during 3 months) 2. Qigong (12 classes, 90 min each, during 3 months)	1. Pain intensity (VAS) 2. Pain (Functional Rating Index)	Participation in a 3-month yoga or qigong program did not improve chronic LBP, back function and quality of life.
Hall et al., 2016	England	102 subjects *M* = 25, *F* = 77 Mean age: 66.5	Chronic non-specific low back pain	NA	G1 (*n* = 51): Tai Chi group G2 (*n* = 51): Wait-list Control group (usual care)	10 weeks	Two 40 min sessions per week for the first 8 weeks, and one 40 min session class for the last 2 weeks	1. Pain intensity (NRS) 2. Pain related disability (RMDQ)	The total effects showed better outcome on measures for the tai chi group and were all significant at the 5% significance level.
Zou et al., 2019a	China	43 subjects *M* = 11, *F* = 32 Mean age: 58	Chronic non-specific low back pain	NA	G1 (*n* = 15): Tai Chi group G2 (*n* = 15): Core stability training group G3 (*n* = 13): Control group (normal daily activities)	12 weeks	Three sessions per week, with each session lasting 60 min for 12 weeks	1. Pain intensity (VAS) 2. Neuromuscular function assessment	Chen-style tai chi and Core stability training were found to have protective effects on neuromuscular function in aging individuals with non-specific LBP, while alleviating non-specific chronic pain.
Phattharasupharerk et al., 2019	Thailand	72 subjects *M* = 26, *F* = 46 Mean age: 35.25	Chronic non-specific low back pain	No medication taken during the period of study	G1 (*n* = 36): Qigong group G2 (*n* = 36): waiting list (general advice)	6 weeks	60 min session per week for 6 weeks	1. Pain intensity (VAS) 2. Back functional disability (RMDQ)	The qigong group showed significant improvement in pain and functional disability both within the group and between groups.
Liu et al., 2019	China	43 subjects *M* = 11, *F* = 32 Mean age: 59	Chronic non-specific low back pain	NA	G1 (*n* = 15): Tai Chi group G2 (*n* = 15): Core stabilization training group G3 (*n* = 13): No intervention	12 weeks	Three 60-min sessions per week for 12 weeks	1. Pain intensity (VAS) 2. Knee and ankle joint position sense	Tai Chi and Core Stabilization training have significant effects on pain VAS but not on joint position sense.
Yao et al., 2020	China	72 subjects *M* = 14, *F* = 58 Mean age (± SD): 53.5 ± 15	Chronic non-specific low back pain	No medication taken during the period of study	G1 (*n* = 36): Wuqinxi group G2 (*n* = 36): General exercise group	24 weeks	Four times a week with 1 h of each session for 24 weeks	1. Pain intensity (VAS) 2. Trunk Muscle Strength	Wuqinxi had better effects on chronic LBP for a long time compared with general exercise, including pain intensity and quality of life.
Ma et al., 2020	China	84 subjects *M* = 59, *F* = 25 Mean age: 36	Axial spondyloarthritis	NA	G1 (*n* = 42): Tai Chi group G2 (*n* = 42): Standard exercise therapy	12 weeks	Three 30–40 min sessions per week for 12 weeks	1. Pain intensity (VAS) 2. Spinal motor function	Compared with standard exercise therapy, “tai chi spinal exercise” has an ideal effect in patients with axial spondyloarthritis, which can more effectively relieve patient’s LBP and improve spinal motor function, with shorter training time and better compliance.
Sherman et al., 2020	USA	57 subjects *M* = 22, *F* = 35 Mean age: 73	Chronic non-specific low back pain	NA	G1 (*n* = 28): Tai Chi group G2 (*n* = 12): Health education group G3 (*n* = 17): Usual care group	12 weeks	Two 60 min sessions per week for 12 weeks	1.0–10-point pain intensity measure 2. Pain related disability (RMDQ)	Compared with health education, tai chi participants rated both the helpfulness of classes and their likelihood of recommending the classes to other significantly higher.

LBP, low back pain; VAS, visual analog scale; NRS, numerical rating scale; RMDQ, Roland-Morris Disability Questionnaire.

## Results

### Search results

**FIGURE 1:**
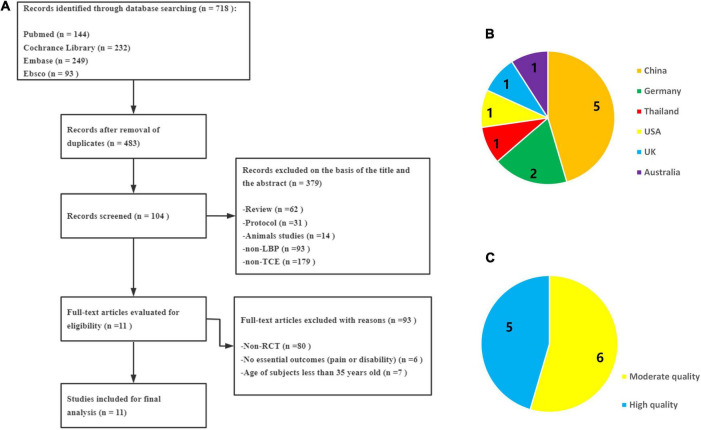
**(A)** A PRISMA flow diagram of the literature screening and selection processes. **(B)** Distribution of countries. **(C)** Quality assessment of PEDro.

As shown in Figure 1A, 718 papers in related fields were retrieved from the four electronic databases. After removing 235 duplicates, 483 articles were screened for eligibility. Through reviewing the titles, abstracts, and full contents of the selected studies, we excluded another 472 articles (review = 62, protocol = 31, animal studies = 14, non-LBP = 93, non-TCE = 179, non-RCT = 80, no essential outcomes = 6, age of subjects less than 35 years old = 7). Finally, 11 RCTs were included in this review (Hall et al., 2011, 2016; Blodt et al., 2015; Teut et al., 2016; Qaseem et al., 2017; Liu et al., 2019; Phattharasupharerk et al., 2019; Zou et al., 2019a; Ma et al., 2020; Sherman et al., 2020; Yao et al., 2020).

### Study characteristics

Table 1 shows the key characteristics of all included RCTs. Eleven RCTs involving 1,256 participants (810 females) ranging in age from 35 to 73 years were included. The sample size of each RCT ranged from 43 to 176. These RCTs were performed in China, Germany, Australia, England, Thailand, and the USA between 2013 and 2020 (Figure 1B). Three kinds of TCE programs were used to treat CLBP in all intervention groups (7 for Tai Chi, 3 for Qigong, and 1 for Wuqinxi). In the control group, active interventions (such as core training, exercise therapy, or yoga) or passive interventions (such as health education) were used. The treatment duration ranged from 6 weeks to 6 months, with each session lasting from 30 to 90 min, of which 12 weeks was the most common in six trials. The visual analogue scale (VAS) and the numerical rating scale (NRS) were used to evaluated the major outcomes for pain severity.

### Quality assessment

Supplementary Table 1 summarizes the quality evaluation results for each RCT by using the PEDro scale. The quality of all 11 studies considered in this review ranged from moderate to high (Figure 1C). In most RCTs, participants, therapists, and assessors were not blinded.

### Effect of traditional Chinese exercise on pain

All 11 included studies involving 1,256 patients with LBP examined the effect of different types of TCE on pain intensity. Ten studies suggested that TCE group outperformed the control group in terms of pain relief, but one of the studies found that the effectiveness of Qigong for pain relief decreased over time. Only one study found no significant difference in pain alleviation between the Qigong and the control groups (Teut et al., 2016).

### Effect of traditional Chinese exercise on pain-related disability, quality of life, and sleep quality

Of the 11 included studies, five studies investigated the effects of TCE on back functional disability, three studies evaluated the effect on quality of life (QOL), and four studies determined the effect on sleep quality and satisfaction. Three studies suggested that Tai Chi had effectively improved back pain-related disability compared with the control group (Hall et al., 2011, 2016; Sherman et al., 2020). Phattharasupharerk et al. and Blödt et al. reported that Qigong did not improve back functional disability effectively because the Roland–Morris Disability Questionnaire (RMDQ) scores did not meet the minimal clinically important difference level (Blodt et al., 2015; Phattharasupharerk et al., 2019). Additionally, Qigong and Wuqinxi had effectively improved QOL and depression compared with the baseline, but no statistical difference was found compared with the control group (Blodt et al., 2015; Teut et al., 2016; Yao et al., 2020). Overall, most studies did not find any difference in the secondary outcomes tested (disability, QOL, and sleep quality).

### Adverse events

Only two studies reported adverse events. Amanda et al. found that four subjects reported a slight increase in back pain at the beginning of Tai Chi training, which was relieved by the third or fourth week of the training (Hall et al., 2011). Blödt et al. found that both the Qigong group (*n* = 10) and exercise group (*n* = 10) reported suspected adverse events (e.g., muscle soreness and tenseness, dizziness, mood fluctuation, and increased back pain) (Blodt et al., 2015).

## Discussion

### Effectiveness of traditional Chinese exercise on middle-aged and elderly patients with chronic low back pain

Pain management in elderly patients with CLBP is particularly challenging, and long-term opioid use was associated with an increased risk of comorbidities (Makris et al., 2014), psychological distress [e.g., depression (Maher et al., 2017)] and other health problems (e.g., falls, osteoporosis, and muscular atrophy) (Chou et al., 2015). A previous review suggested that people who experienced LBP had a higher risk of recurrence (Taylor et al., 2014). Therefore, current guidelines recommend that treatment should focus on reducing pain and its associated dysfunction, and exercise is an effective treatment option (Koes et al., 2010; Zhang et al., 2020). This systematic review included 11 RCTs involving 1,256 patients with CLBP aged over 35 years to assess the overall effect of TCE in middle-aged and elderly patients with CLBP. Our results indicated that TCE could be an effective therapy for reducing pain and improving function in the patients. All treatments (Tai Chi, Qigong, and Wuqinxi) showed positive effects compared with baseline measurements.

Pain intensity relates to the degree to which a person is harmed by CLBP and can be quantified to estimate the severity of pain (Ostelo and de Vet, 2005). The results from this systematic review suggest that TCE significantly reduce the VAS or NRS scores of patients with CLBP. Compared with the control and exercise therapy groups, TCE showed better effects in alleviating pain, which is consistent with prior reviews on other exercise therapies (Hayden et al., 2005; Macedo et al., 2013). Only one study showed that 3 months of yoga or Qigong training had no effect on back pain, back function, or QOL in older patients with CLBP (Teut et al., 2016). However, the number of studies was insufficient to conclude every type of TCE. Additionally, several RCTs showed that Tai Chi/Qigong significantly contributed to proprioception and neuromuscular function in the lower limbs (Zou et al., 2019a). An RCT involving 84 patients showed that Tai Chi was more effective in improving spinal movement function, with shorter training time and better compliance compared with standard exercise therapy. Another RCT showed that office workers with CLBP achieved better health behavior after 6 weeks of Qigong exercise as well as significantly improved mental state, back function, range of motion, and core muscle strength (Phattharasupharerk et al., 2019). Despite the negative findings of some studies, the fact that CLBP is difficult to manage suggests that TCE could be a possible option for managing pain in middle-aged and elderly patients with CLBP.

### Underlying mechanisms of traditional Chinese exercise for improving chronic low back pain

TCE focuses on the integration of mental regulation, breathing, and movement control in addition to internal energy regulation (Zou et al., 2019b).

First, as a foundation of mind–body interaction, meditation and rhythmic breathing can effectively boost vitality and induce energy to flow through the body, which in turn drives body movement to alleviate pain (Zou et al., 2017b). Self-awareness combined with self-correction of posture and movement of the body, flow of breath, and mental stilling activates natural self-regulatory (self-healing) abilities and stimulates a balanced release of endogenous neurohormones and a variety of natural health recovery mechanisms (Jahnke et al., 2010; Linek et al., 2020). Multiple elements of health, including mood, pain, immunity, and peripheral autonomic nervous system function, can be regulated by concentration and mindful meditation (Wayne and Kaptchuk, 2008). Some TCE programs use meditation and imagination to guide and distract attention away from pain, which can help reduce pain and enhance psychosocial health. Evidence indicates that poor pain-related outcomes (e.g., pain levels and disability) have been linked to a higher level of pain catastrophizing (Peng, 2012). Pain-related catastrophizing is a negative cognitive response to pain. For example, Hall et al. found that Tai Chi could help with pain-related symptoms by changing cognitive appraisal results, such as lowing catastrophic outcomes (Hall et al., 2016). Additionally, a recent meta-analysis suggested that adults with chronic diseases obtained reduced muscle pain through mindfulness-based training (Zou et al., 2018b).

Second, TCE relieves pain by combining muscle strength, static balance, and dynamic balance, and these concepts are quite similar to other pain-relieving therapies, such as core stabilization training (Hall et al., 2011; Gordon and Bloxham, 2016). TCE can help relieve back pain by strengthening lumbar muscles and improving pelvic–lumbar neuromuscular function and proprioception. Qigong, Tai Chi, and Wuqinxi involve a series of slow, flowing, dance-like body movements. In particular, combining slow coordinated postures can transfer upper and lower body momentum and achieve balance depending on continuous squatting and weight shifting on both legs throughout the exercise (Zou et al., 2018b). Improvements in lower limb function and lumbar flexibility improved CLBP-related physical activities (i.e., sitting and standing, stair climbing, and walking) (Masharawi and Nadaf, 2013). Compared with taking anti-osteoporosis drugs, Wuqinxi can significantly reduce pain symptoms and increase the bone density of lumbar vertebrae, suggesting the positive effect of Wuqinxi on CLBP (Wei et al., 2015). Our previous work also suggested that Chen-style Wai Chi had protective effects on neuromuscular function in elderly patients with CLBP while relieving non-specific chronic pain (Zou et al., 2019a).

In addition to correcting postural control and enhancing muscle strength to relieve back pain, the pain-relieving effects of TCE may be linked to changes in TCE-induced brain activity. Long-term Tai Chi practice resulted in an increase in cortical thickness of the inferior segment of the circular sulcus of the insula as well as a decrease in the functional homogeneity of the left anterior cingulate cortex (ACC) (Wei et al., 2013, 2014). ACC plays a crucial role in the emotional aspects of pain (Fuchs et al., 2014; Barthas et al., 2015). Inhibiting ACC may help alleviate chronic pain (Gu et al., 2015). The improvement of ACC functional specificity after Tai Chi training may contribute to pain relief, thereby explaining its analgesic effect. An increase cortical thickness of insula observed in long-term Tai Chi practitioners may also contribute to pain relief through better processing of pain-related cognitive information. To uncover the neurological mechanism underlying TCE-mediated pain relief, further studies should be conducted into the direct relationship between pain perception and TCE-mediated alterations in these brain regions.

This systematic review has some limitations. First, it has location and language bias, with five studies from China, two studies from Germany, and only one study from Thailand, the USA, the UK, and Australia, which were all published in English. Second, the TCE intervention differed greatly in terms of exercise type (Tai Chi, Qigong, Wuqinxi), duration (6–24 weeks), frequency (2–5 times/week), and control group. In the future, a detailed categorization of different types of TCE programs and controls will be required. Third, most of the included RCTs did not adopt blind methods (subject, therapist, and assessor blinding), which might lead to biased subjective expectations and exaggerate the research findings. Finally, most RCTs employed followed up for few months only, so the long-term efficacy of TCE in patients with CLBP remains unclear.

## Conclusion

This systematic review shows that TCE is beneficial in relieving pain and improving pain-related dysfunction for middle-aged and elderly patients suffering from CLBP. As a convenient, cost-effective therapy with few adverse events, TCE could be recommended for elderly patients with CLBP. Nevertheless, the long-term efficacy of TCE in elderly patients with CLBP must be assessed, and theories on how TCE could treat and prevent CLBP require further investigation. In the future, more controlled studies with larger scale and stricter quality should be conducted to explore the long-term efficacy of TCE in elderly patients with CLBP.

## Author contributions

X-QW and H-YX: software, formal analysis, data curation, writing – original draft preparation, visualization, and project administration. LH: conceptualization, methodology, validation, investigation, resources, supervision, and funding acquisition. S-HD and Q-HY: methodology, validation, and writing – review and editing. All authors contributed to the manuscript revision, read, and approved the submitted version.

## Footnotes

1
https://osf.io/nwgsf
