# Supplementary material for: The effect and mechanism of traditional Chinese exercise for chronic low back pain in middle-aged and elderly patients: A systematic review
Source: Front Aging Neurosci. 2022 Oct 10;14:935925. doi: 10.3389/fnagi.2022.935925 (PMC9590689; doi:10.3389/fnagi.2022.935925)
Supplement: Supplementary file 1 [file Table_1.DOCX]

| Supplementary table 1. Physiotherapy Evidence Database Scores of Included Studies | | | | | | | | | | | | |
| --- | --- | --- | --- | --- | --- | --- | --- | --- | --- | --- | --- | --- |
| Article, Year | Random allocation | Concealed allocation | Baseline comparability | Blind subjects | Blind therapists | Blind assessors | Adequate follow-up | Intention-to-treat analysis | Between-group comparisons | Point estimates and variability | Total (0-10 Scale) | Quality |
| Hall(2011) | Yes | Yes | Yes | No | No | No | Yes | Yes | Yes | Yes | 7 | High |
| Wu(2013) | Yes | No | Yes | No | No | Yes | No | No | Yes | Yes | 5 | Moderate |
| Blodt (2015) | Yes | Yes | Yes | No | No | No | Yes | Yes | Yes | Yes | 7 | High |
| Teut (2016) | Yes | Yes | Yes | No | No | No | Yes | Yes | Yes | Yes | 7 | High |
| Hall(2016) | Yes | Yes | Yes | No | No | No | No | Yes | Yes | Yes | 6 | Moderate |
| Zou (2019) | Yes | No | Yes | No | No | Yes | Yes | No | Yes | Yes | 6 | Moderate |
| Phattharasupharerk(2019) | Yes | Yes | Yes | No | No | Yes | Yes | Yes | Yes | Yes | 8 | High |
| Liu (2019) | Yes | No | Yes | No | No | No | Yes | No | Yes | Yes | 5 | Moderate |
| Yao(2020) | Yes | Yes | Yes | No | No | Yes | Yes | No | Yes | Yes | 7 | High |
| Ma (2020) | Yes | No | Yes | No | No | No | Yes | No | Yes | Yes | 5 | Moderate |
| Sherman (2020) | Yes | Yes | Yes | No | No | No | Yes | No | Yes | Yes | 6 | Moderate |
| High quality: total score ≥ 7; moderate quality: total score 4 - 6; low quality: total score ≤ 3. Scoring: 0 = no or unable to determine; 1 = yes. | | | | | | | | | | | | |
