# Supplementary material for: The effect and mechanism of traditional Chinese exercise for chronic low back pain in middle-aged and elderly patients: A systematic review
Source: Front Aging Neurosci. 2022 Oct 10;14:935925. doi: 10.3389/fnagi.2022.935925 (PMC9590689; doi:10.3389/fnagi.2022.935925)
Supplement: Supplementary file 2 [file Table_2.DOCX]

**Supplementary material 1. The detailed search strategies for all databases**

1. **Search Strategy for Pubmed**

#1. tai chi[Title/Abstract] OR "taiji*"[Title/Abstract] OR qigong[Title/Abstract] OR liuzijue[Title/Abstract] OR wuqinxi[Title/Abstract] OR yijinjing[Title/Abstract] OR baduanjin[Title/Abstract] OR "traditional exercise"[Title/Abstract] OR traditional chinese medicine[Title/Abstract] OR"chinese traditional exercise"[Title/Abstract] OR "traditional chinese exercise"[Title/Abstract] OR "chinese exercise"[Title/Abstract]

#2. back pain[Title/Abstract] OR low backache[Title/Abstract] OR lower back pain[Title/Abstract] OR lumbago[Title/Abstract] OR Lumbosacral pain[Title/Abstract] OR Sciatica[Title/Abstract]

#3. Searched by #1 AND #2

1. Search results: 144 articles
2. Timespan: 1999.01.01-2022.3.19
3. Retrieval time: 2022.03.19
4. **Search Strategy for Embase**

#1. ‘tai chi’:ti,ab OR "taiji*":ti,ab OR qigong:ti,ab OR liuzijue:ti,ab OR wuqinxi:ti,ab OR yijinjing:ti,ab OR baduanjin:ti,ab OR "traditional exercise":ti,ab OR ‘traditional chinese medicine’:ti,ab OR "chinese traditional exercise":ti,ab OR "traditional chinese exercise":ti,ab OR "chinese exercise":ti,ab

#2. ‘back pain’:ti,ab OR ‘low backache’:ti,ab OR ‘lower back pain’:ti,ab OR ‘lumbago’:ti,ab OR ‘Lumbosacral pain’:ti,ab OR Sciatica:ti,ab

#3. Searched by #1 AND #2

1. Search results: 232 articles
2. Timespan: 1999.01.01-2022.3.19
3. Retrieval time: 2022.03.19
4. **Search Strategy for Cochrane Library**

#1. ‘tai chi’:ti,ab OR "taiji*":ti,ab OR qigong:ti,ab OR liuzijue:ti,ab OR wuqinxi:ti,ab OR yijinjing:ti,ab OR baduanjin:ti,ab OR "traditional exercise":ti,ab OR ‘traditional chinese medicine’:ti,ab OR "chinese traditional exercise":ti,ab OR "traditional chinese exercise":ti,ab OR "chinese exercise":ti,ab

#2. ‘back pain’:ti,ab OR ‘low backache’:ti,ab OR ‘lower back pain’:ti,ab OR ‘lumbago’:ti,ab OR ‘Lumbosacral pain’:ti,ab OR Sciatica:ti,ab

#3. Searched by #1 AND #2

1. Search results: 249 articles, 211 trails
2. Timespan: 1999.01.01-2022.3.19
3. Retrieval time：2022.03.19
4. **Search Strategy for CINAHL (Ebsco)**

S1. AB(tai chi OR taiji OR qigong OR liuzijue OR wuqinxi OR yijinjing OR baduanjin OR traditional exercise OR chinese traditional exercise OR traditional chinese exercise OR chinese exercise)

S2. TI(tai chi OR taiji OR qigong OR liuzijue OR wuqinxi OR yijinjing OR baduanjin OR traditional exercise OR chinese traditional exercise OR traditional chinese exercise OR chinese exercise)

S3. S1 OR S2

S4. AB(‘back pain’ OR ‘low backache’ OR ‘lower back pain’ OR ‘lumbago’ OR ‘Lumbosacral pain’ OR Sciatica

S5. TI(‘back pain’ OR ‘low backache’ OR ‘lower back pain’ OR ‘lumbago’ OR ‘Lumbosacral pain’ OR Sciatica)

S6. S4 OR S5

S7. Searched by S3 AND S6

1. Search results: 93 articles
2. Timespan: 1999.01.01-2022.3.19
3. Retrieval time: 2022.03.19
